# Viral Ubiquitin and Ubiquitin-Like Deconjugases—Swiss Army Knives for Infection

**DOI:** 10.3390/biom10081137

**Published:** 2020-08-01

**Authors:** Maria Grazia Masucci

**Affiliations:** Department of Cell and Molecular Biology, Karolinska Institutet, S-17177 Stockholm, Sweden; maria.masucci@ki.se

**Keywords:** ubiquitin-like deconjugase, herpesvirus, coronavirus, virus cycle, innate immunity, type I IFN

## Abstract

Posttranslational modifications of cellular proteins by covalent conjugation of ubiquitin and ubiquitin-like polypeptides regulate numerous cellular processes that are captured by viruses to promote infection, replication, and spreading. The importance of these protein modifications for the viral life cycle is underscored by the discovery that many viruses encode deconjugases that reverse their functions. The structural and functional characterization of these viral enzymes and the identification of their viral and cellular substrates is providing valuable insights into the biology of viral infections and the host’s antiviral defense. Given the growing body of evidence demonstrating their key contribution to pathogenesis, the viral deconjugases are now recognized as attractive targets for the design of novel antiviral therapeutics.

## 1. UbL Signaling Networks

Viruses have shaped the fate of human societies throughout history. Understanding how these potentially life-threatening pathogens establish infection and how they interact with their hosts is our best strategy for acquiring the means to control the diseases they cause. Being obligatory intracellular parasites, viruses face a double challenge. On one side, they need to commandeer the molecular machinery of the host cell to support the production of new virus particles, while on the other side, they must hold back the multifaceted cellular and organismal defenses that are triggered by infection. These challenges are met by the expression of specialized viral products that hijack or manipulate critical cellular functions. Many of these viral pathogenicity factors are multifunctional proteins that mimic the activity of cellular counterparts whose activity controls key aspects of normal cell physiology. 

Virtually all cellular processes are regulated by posttranslational modifications that dictate the function, subcellular localization, and interactions of effector proteins. Among those, the covalent attachment of small polypeptides of the ubiquitin family (henceforth collectively referred to as ubiquitin-like polypeptides, UbLs) provides a flexible means to control the activity and fate of the modified substrate. Cell functions that orchestrate the outcome of infection such as the cell cycle, cell survival and programmed cell death, gene expression, protein trafficking and degradation, autophagy, and the immune response are all dependent on UbL modifications [1]. It is therefore not surprising that viruses have evolved means to interfere with the UbL signaling networks in order to secure a cellular environment conducive to their own replication and spread. 

**Figure 1:**
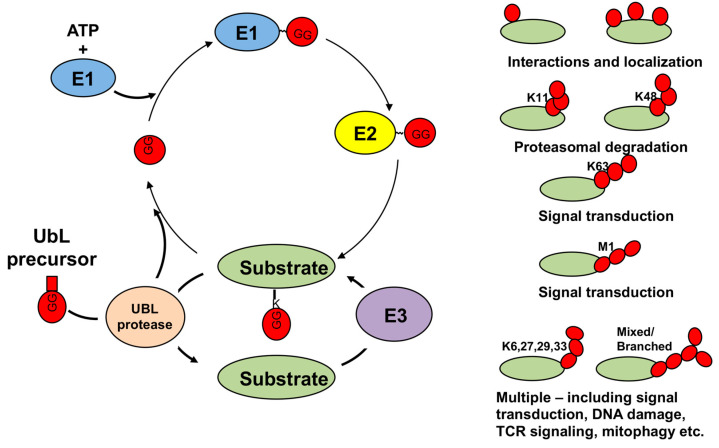
Schematic illustration of the UbL activation, conjugation and deconjugation cycle. The covalent attachment of UbLs to their substrates involves sequential catalytic reactions that initiate with processing of the UbL precursor by a specific UbL protease. The mature UbL is activated by an activating enzyme (E1) and then transferred to a conjugating enzyme (E2) that, with the help of a substrate-specific ligase (E3), transfer the activated UbL to the ε-amino residue of a Lys on the target protein via a covalent isopeptide bond. Additional UbLs can be linked to the previous one to form chains. UbL-specific proteases can reverse the modification, supplementing the cellular pools of free UbLs. The attachment of a Ub moiety to the N-terminal Met1 or to an internal Lys residue of the previous Ub (K6, K11, k27,K29,K33,K48 or K63) results in the formation of topologically different poly-Ub chains that, upon recognition by signal transducers contain dedicated binding domains, target the substrates various fates and cellular functions

The UbLs are a family of structurally related small polypeptides that share a β-grasp fold organization consisting of a mixed β-sheet structure with a central α-helix [2]. To date, seventeen human UbLs have been reported to be conjugated to other molecules. In addition to ubiquitin (Ub), the UbLs include the small ubiquitin-related modifier (SUMO)-1, -2, and -3; NEDD8 (neural precursor cell expressed, developmentally downreagulated-8); ISG15 (interferon stimulated gene-15); UFM1 (ubiquitin-fold modifier-1); URM1 (ubiquitin-related modifier-1); FAT10 (HLA-F adjacent transcript 10); MNSFβ (monoclonal nonspecific suppressor factor β); and the LC3 (microtubule-associated light chain-3) and GABARAP (γ-aminobutyric acid receptor associated protein) family of modifiers [3]. The attachment of UbLs to their protein or, in the case of the LC3/GABARAP family, lipid substrates is mediated by an enzymatic cascade that starts with processing of the UbL precursor by a specific protease, which generates a C-terminal Gly residue required for conjugation. The mature UbL becomes the substrate of an activating enzyme (E1) that forms a high-energy thiolester bond with the C-terminal Gly and then loads the activated UbL on the catalytic Cys residue of a conjugating enzyme (E2). The E2 transfers the UbL to the substrate with the help of a ligase (E3) that promotes the transfer [4,5] (Figure 1). As a rule, each UbL conjugation system involves distinct sets of dedicated E1, E2, and E3, enzymes but enzyme sharing is not uncommon and E3 ligases with mixed specificity for Ub and ISG15, e.g., TRIM25 (Tripartite motif-25) [6]; ubiquitin and NEDD8, e.g., MDM2 (Mouse double minie-2) [7]; or Ub and SUMO, e.g., TOPORS (TOP1 binding Arginine/Serine rich protein), TRAF7 (TNF receptor associated factor-7), UHRF2 (Ubiquitin like with PHD and RING finger domain-2), and TRIM27 [8,9,10,11] have been described. 

**Figure 2:**
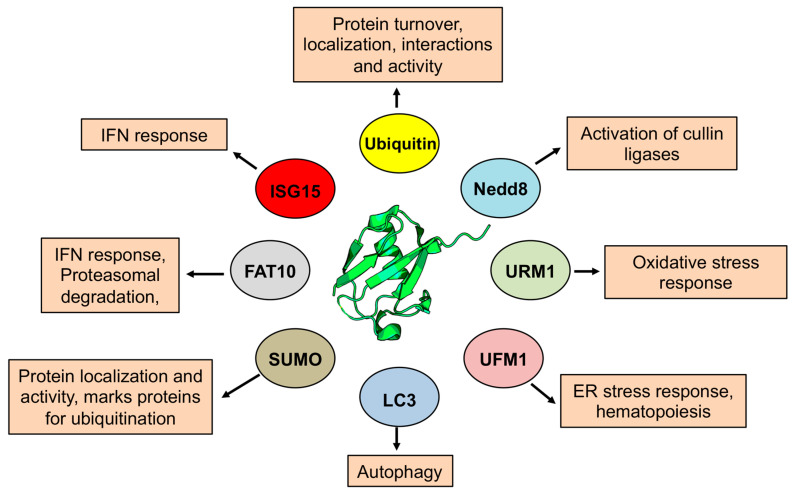
Diverse functions of the UbLs. The UbLs are involved in a large variety of cellular processes, with each UbL targeting distinct functions depending on the range of modified substrates. The best-known function of ubiquitin is the marking of substrates for degradation by the proteasome, but different types of ubiquitination regulate endocytosis protein trafficking, transcription and translation, cell signaling, histone modification and DNA repair. Other UbL have similar but usually more restricted roles in the regulation of cellular functions. SUMOylation is involved in the formation of protein complexes that regulate transcription, DNA repair different stress responses and can also mark proteins for ubiquitin-dependent degradation. NEDD8 is best known for its role in regulating the activity of cullin-RING ligases, which in turn regulates substrate degradation. UbLs, of the LC3/GABARAP family are involved in the process of autophagy.

Ubiquitin is the first recognized and best-known member of the family. The covalent attachment of Ub, ubiquitination, is mediated by specific combinations of E2s and E3s that promote the formation of a peptide bond between the C-terminal Gly and the N-terminal Met or the ε-amino group of a Lys residue in the substrate. In addition to the attachment of a single Ub to one (mono-Ub) or several (multi-Ub) Lys residues, poly-Ub chains can be formed upon attachment of a new Ub moiety to Met1 or Lys6, 11, 27, 29, 33, 48, or 63 of the first conjugated Ub. In the poly-Ub chain, Ub is usually attached to the same Lys residue on each Ub in the chain but mixed-linkage and branched poly-Ub chains may be more common than originally thought [12] (Figure 1). The attachment of Ub or poly-Ub chains generates new interaction surfaces in the modified substrate that are recognized by signal transducers via dedicated ubiquitin binding domains [13]. Signal transducers that recognize other UbLs have their own specific binding motifs resulting in a broad spectrum of distinct signals that engage the modified substrate in specialized functions (Figure 2). For example, Lys48-linked poly-Ub chains (K48poly-Ub) usually target the substrate for degradation by the proteasome whereas K63poly-Ub have non-proteolytic functions, often related to protein localization and protein–protein interactions [14]. Mono- or poly-SUMOylation regulates protein localization and the formation of protein complexes involved in DNA replication and stress responses [15]. Poly-SUMO chains may also serve as a signal for Ub-dependent proteasomal degradation following recognition by specialized E3 that carry multiple SUMO-interacting motifs [16]. The activation of cullin-RING ligases (CRLs) by NEDDylation of the cullin scaffold provides another example of cross-talk between different types of UbL modification [17]. Other UbLs play important roles in different types of stress responses as illustrated by involvement of URM1 and UFM1 in the regulation of oxidative stress [18,19] and ER stress [20,21], respectively, and by the involvement of FAT-10 [22] and ISG15 [23,24] in the cellular and immune response to infection. The conjugation of UbLs of the LC3/GABARAP family to the membrane lipid phosphatidylethanolamine underlies their involvement in the expansion and fusion of autophagic membranes [25].

The conjugation of UbLs is highly dynamic and reversible, allowing for the fine-tuning and rapid remodeling of signal transduction pathways in response to different stimuli. Deconjugating enzymes catalyze the removal of the UbLs from their substrates, resulting in either complete loss or editing/trimming of the UbL chain. In humans, around hundred Ub-specific deconjugases (also called deubiquitinases, DUBs), belong to families that differ in structure and catalytic mechanisms [26,27]. 

The majority are cysteine proteases containing an active-site catalytic triad composed of a Cys nucleophile and closely situated His and Asp/Asn residues. The Cys protease families include Ub-specific proteases (USPs), ubiquitin C-terminal hydrolases (UCH), ovarian tumor domain proteases (OTUs), Machado–Josephin disease proteases (MJD), motif interacting with ubiquitin novel DUB family (MINDY), and Zinc finger with UFM1-specific peptidase domain protein (ZUFSP), whereas the JAB1/MPN/MOV34 (JAMM) deconjugases are zinc-dependent metalloproteases. Members of the USP family generally cleave all Ub linkage types without a clear preference [28], while the members of the OTU enzymes are often linkage specific [29]. As a rule, different UbLs have their own sets of specific deconjugases. However, several Ub deconjugases exhibit promiscuous activity against ISG15 or NEDD8 [30,31], probably due to the closely similar C-terminal tail region of these UbLs. 

## 2. UbLs in Viral Infection

In view of their limited genome size, viruses need to co-opt the host-cell machineries for virtually all aspects of their life cycle. Hence, UbL-regulated cellular functions are exploited for viral entry, transcription and replication of the viral genomes, synthesis of viral proteins, and assembly of new virions and for the maturation and exit of viral particles from the infected cell [32,33,34,35,36]. In addition, UbLs play key roles in the regulation of the innate and adaptive immune defense that counteract infection. A large number of UbL ligases and deconjugases participate in different aspects of the antiviral immune response, ranging from the signaling of viral nucleic acid sensors in innate immunity and inflammation to the maturation of antigen presenting cells and the activation of antigen-specific T-cell responses [37,38]. 

**Figure 3:**
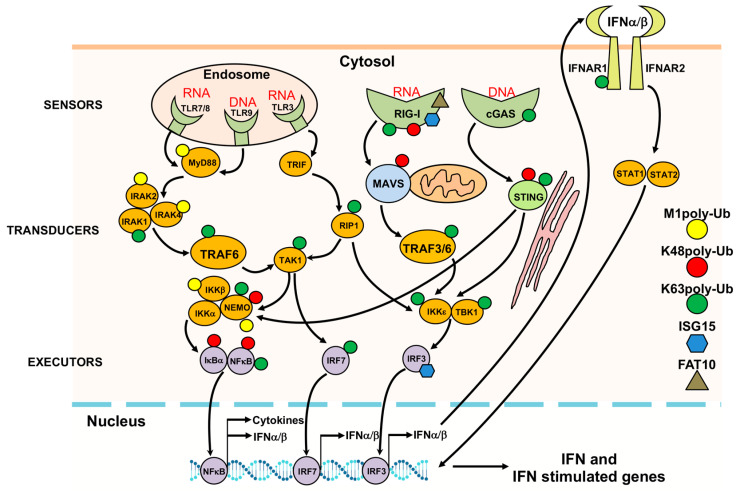
Regulation of the antiviral response by UbLs. The covalent or non-covalent attachment of different type of poly-Ub chains, ISG15 and FAT10 to viral nucleic acid sensors, signal transducers and executors regulates the innate immune response. UbL modifications are indicated by symbols of different color and shape attached to the proposed or validated substrates.

The pleiotropic role of UbLs in orchestrating the antiviral defense is well illustrated by their contribution to the activation and fine-tuning of the early response to infection [39] (Figure 3). The recognition of incoming viral genomes by DNA or RNA sensors located in endosomes (Toll-like receptors, TLRs) or in the cytosol of the infected cells (including the retinoic acid inducible gene, RIG-I-like receptors, RLRs, and cytoplasmic DNA sensors such as cyclic GMP-AMP synthase, cGAS) leads via a cascade of signal transduction events to the activation of executor transcription factors that drive the synthesis of antiviral molecules such as type I and II interferons (IFNs) and pro-inflammatory cytokines [40,41]. In turn, binding of IFNs to their specific receptors activates a new signaling cascade that leads to transcription of IFN-sensitive genes for which the products mediate establishment of an antiviral state [42]. E3 ligases regulate these signaling pathways via the attachment of different types of polyubiquitin chains and UbL polypeptides [43,44]. For example, the viral nucleic acid sensor RIG-I is activated by K63-polyubiquitination mediated by the ligases TRIM4 [45], Riplet [46], and TRIM25 [47], while signaling is terminated by K48-polyubiquitination mediated by the Ring Finger protein 125 (RNF125) ligase [48], which promotes proteasomal degradation. The attachment of M1- or K63-polyubiquitin chains to signaling mediators such as IRAK1 (Interleukin 1 associated kinase-1) [49,50], TRAF6 [51], RIP1 (Receptor interacting protein-1) [52], TRAF3 (TNF receptor associated factor-3) [53], MAVS (Mitochondrial antiviral signaling protein) [54], NEMO (NF-κB essential modulator) [55], and STING (Stimulator of IFN genes) [56] promotes activation of the kinases IKK (IκB kinase), TAK1 (Transforming growth factor beta activated kinase-1), and TBK1 (TANK binding kinase-1) [57,58] that phosphorylate the executor transcription factors NF-κB (Nuclear factor-κB, IRF3 (Interferon regulatory factor-3), and IRF7, leading to their activation and nuclear translocation. Phosphorylation may also serve as a signal for ubiquitination as illustrated by the phosphorylation-dependent K48-polyubiquitination of IκBα by the βTRCP E3 ligase, which leads to degradation of the inhibitor and activation of NF-κB [59]. Other types of UbL modifications exert similar regulatory functions. Thus, ISGylation targets RIG-I for degradation by autophagy, reducing the levels of both basal and virus-induced IFN promoter activity [60], while ISGylation of IRF3 by the HERC5 (HECT and RLD domain containing-5) ligase was shown to promote sustained signaling by protecting IRF3 for ubiquitination and proteasomal degradation [61]. FAT10 was shown to form an inhibitory complex with RIG-I, leading to the formation of insoluble RIG-I aggregates, which prevented the translocation of RIG-I to MAVS and halted signaling [62]. Neddylation of MyD88 was shown to negatively regulate NF-κB signaling by antagonizing its ubiquitination [63], while SUMOylation of RIG-I, IRF3, and IRF7 was shown to affect both their stability and signaling properties [64].

The production of type I IFN leads to transcriptional activation of numerous IFN stimulated genes (ISGs) whose products cooperate in the establishment of an antiviral state in both the infected and adjacent cells [65]. ISG15 and its conjugation enzymes are strongly upregulated by IFN, and hundreds of putative targets of ISGylation have been identified by mass spectrometry analysis although only a few have been experimentally validated [66]. The conjugation of ISG15 to both viral and cellular proteins was shown to impair virus replication and spread [67,68]. Thus, the ISGylation of de novo synthesized viral proteins may hinder their interaction with host proteins that are required for replication, may disrupt their catalytic function, or may alter the oligomerization of capsid proteins leading to a decrease in the number and infectivity of virus particles [69,70,71]. In addition, ISGylation inhibits the function of cellular proteins that regulate vesicular trafficking and are required for virus budding and release, including components of the endosomal sorting complex required for transport (ESCRT) [72,73]. Recent studies suggest that ISG15 may participate in the regulation of herpesvirus latency. Numerous ISGs were strongly upregulated in primary human oral fibroblasts latently infected with Kaposi’s sarcoma-associated herpesvirus (KSHV) and in KSHV-positive primary effusion lymphoma cells, while knockdown of ISG15 or the ISG15 ligase HERC5 induced virus reactivation and the release of infectious virus [74,75]. It should be noted that conjugation-independent functions of ISG15 may also contribute to the control of infection. High serum levels of unconjugated ISG15 have been detected in patients treated with interferon and in mice infected with different viruses [76]. Furthermore, extracellular ISG15 was shown to function as a cytokine with immune modulatory activity [77] and as a chemotactic factor for neutrophils [78], pointing to a possible function of soluble ISG15 in the modulation of inflammatory responses. 

## 3. Viral UbL Deconjugases

Interfering with UbL-dependent processes through deconjugation is a powerful strategy used by viruses to regulate many cellular functions that contribute to or counteract infection. Common means of regulation involve altering the expression of host deconjugases or redirecting the activity of the cellular enzymes towards new cellular or viral substrates [79,80]. In addition, many viruses encode their own deconjugases and increasing evidence supports the involvement of these viral enzymes in the control of infection [81,82,83]. Significant effort has been devoted to uncovering the substrates and cellular functions targeted by viral deconjugases. However, caution should be used in the interpretation of the increasing body of data since many experiments have relied on the overexpression of recombinant viral proteins or isolated enzymatic domains that, outside of the physiological context of infection, often exhibit very potent and broad deconjugase activity. Indeed, temporal and spatial constraints operating in the infected cells are likely to determine the accessibility of a given substrate, while other viral factors expressed during infection may influence substrate specificity. Further complications arise when the viral enzyme exerts both deconjugase and protease activity, as observed for the enzymes encoded by RNA viruses. While some of these caveats can be addressed by sophisticated technologies, including structure determination and powerful mass spectrometry, the use of recombinant viruses expressing catalytically dead mutants of the enzymes has in several cases provided conclusive evidence on the cellular functions targeted during infection and reliable information on the putative substrates.

Both DNA and RNA viruses were shown to encode proteins with UbL deconjugase activity (Table 1), and bioinformatics analysis coupled with in vitro enzymatic assays suggest that the largest viruses may even contain more than one deconjugase, as exemplified by the identification of three bona-fide DUBs in the genome of Epstein-Barr virus (EBV) [84,85]. 

Sequence- and structure-based comparisons with eukaryotic UbL-specific protease families have provided interesting clues on the origin and biology of the viral enzymes. In contrast to the eukaryotic enzymes, the deconjugases encoded by viruses usually target more than one UbL. For example, the adenovirus encoded adenain cleaves Ub and ISG15 conjugates but shares structural similarities with the ubiquitin C-terminal hydrolase UCHL3 and with ULP/SENP-like proteases that cleave SUMO and NEDD8 [86]; the papain-like proteases encoded by coronaviruses (CoV) that are structurally related to mammalian DUBs such as USP7 and USP14 show specificity for Ub and ISG15 and possibly NEDD8 [110,111,112]. Ub and ISG15 conjugates are also recognized by the OTU-like proteases encoded by several animal RNA viruses although this double specificity is not observed in their mammalian counterparts [113]. While the structural similarities point to common ancestry, adaptation mechanisms operating in the context of infection may have selected for variants with broader specificity, which could counterbalance the limited coding capacity of the viral genomes. Based on tertiary fold and architecture of the catalytic triad, the deconjugases encoded by herpesviruses constitute a unique family of enzymes that belong to the papain protease superfamily but are only distantly related to known cellular DUBs [114]. These enzymes cleave with comparable efficiency Ub and NEDD8 conjugates but fail to recognize ISG15 [85,90], pointing to a distinct set of substrates and targeted cellular functions.

A common feature of the viral deconjugases is the embedding of catalytic domains in large multidomain proteins that play pleiotropic roles in infection. For example, the CoV-encoded deconjugases are contained within a relatively well-conserved approximately 20 kD region of the membrane anchored nonstructural protein-3 (Nsp3) [115]. The multidomain Nsp3 is the largest protein encoded by the CoV genome, with an average molecular mass of about 200 kD and an essential component of the replication/transcription complex. Although the domain organization differs between CoV genera due to duplication or absence of some domains, eight domains are found in all known Nsp3. These include, in addition to the deconjugase domain, two ubiquitin-like domains, a catalytically active ADP-ribose-1-phosphatase domain that may play a role during synthesis of viral sub-genomic RNAs, a nucleic acid binding domain with chaperone function, and other less characterized domains including an ER luminal Zn finger domain [116]. Via interaction with other Nsps, Nsp3 scaffolds the assembly of the replicase complex that utilizes ER membranes to organize a microenvironment where the genome replication and transcription machinery is localized. In a similar fashion, the deconjugases encoded by different herpesviruses are located in an approximately 25 kD N-terminal domain of the 250–350 kD large tegument proteins [90]. The function of the large tegument proteins is only partially understood. Studies on the Herpes simplex virus (HSV) encoded member of the family, UL36/VP1-2, show that, via binding to the viral capsid protein UL25 [117] and inner tegument protein UL37 [118], UL36 promotes the transport of a viral DNA loaded capsid along microtubules to the sites of secondary envelopment in the trans-Golgi network [119]. Deletion of UL36 results in failure to fully assemble infectious virus particles [120]. This role in virus assembly is shared by all members of this protein family [101,121] and is independent on the deconjugase function [118]. In addition, upon de novo infection, UL36 guides the transport of incoming semi-uncoated capsids to the nuclear pore, where the viral genome is discharged into the nucleoplasm for viral transcription and replication [122]. Interestingly, cleavage of the N-terminal domain of UL36 that contains the deconjugase activity is required for the release of viral DNA into the nucleus [123].

A feature that distinguishes viral enzymes from their eukaryotic counterparts is the double function as UbL deconjugases and endopeptidases that play key roles in the virus cycle by processing viral proteins. The adenovirus protease (AVP), adenain, is incorporated into immature virus particles, where it becomes activated by forming a thiol bond with an eleven-residue cleavage product of the capsid protein pVI (pVIc) [124]. The activated enzyme cleaves several viral capsid precursor proteins via recognition of the (M/I/L)XGX-G and (M/I/L)XGG-X sequence motifs [87,109]; Proteolytic maturation promotes the assembly of entry-competent viruses and primes the incoming virus particles for uncoating, which is essential for infectivity [125]. Interestingly, binding to the activating peptide induces preferential cleavage at the GX-G site that is overrepresented in the viral proteins [126], suggesting that the substrate repertoire of adenain may change during different phases of infection depending on availability of the activating peptide. In a similar fashion, the CoV papain-like protease (PLpro) contained in Nsp3 cooperates with the major chymotrypsin-like protease (3CLpro) in processing of the viral polyprotein to give rise to the sixteen Nsps that form the viral replicase complex [127]. PLpro recognizes the sequence LXGG at the Nsp1/2, Nsp2/3, and Nsp3/4 boundaries that is identical to the C-terminal sequence of Ub, ISG15, and NEDD8 [110,128]. Processing of the polyprotein by PLpro is required for virus replication, which highlights the essential role of the enzyme in the virus life cycle. Analysis of the crystal structure of PLpro bound to Ub-aldehyde and models of the interaction with Ub-chains and ISG15 revealed a likely mechanism for discrimination between the UbL-conjugates and viral substrates. The recognition of poly-Ub chains and ISG15 was shown to be dependent on simultaneous engagement of two binding domains, S1 and S2, on the surface of PLpro [112]. Mutation of the distal S2 domain significantly impaired the processing of ISG15 and poly-Ub conjugates but did not affect the activity of the protease against the viral polyprotein [129]. 

Given the importance of coronaviruses, adenoviruses, and herpesviruses for human diseases, the next sections will be focused on the deconjugases encoded by these viruses, with particular emphasis on the affected cellular functions and validated substrates. Comprehensive reviews on the UbL deconjugases of animal viruses, including the very interesting family of OUT-domain containing proteases encoded by nairoviruses, were recently published [82].

### 3.1. Coronavirus Deconjugases

Coronaviruses are enveloped viruses with positive-sense, single-stranded RNA (ssRNA) genomes [130]. The first human CoV was identified in the 1960s and was recognized as a causative agent of the common cold [131]. Since then, highly pathogenic CoVs causing severe acute respiratory syndrome (SARS-CoV) [132,133], Middle East respiratory syndrome (MERS-CoV) [134], and the ongoing CoV-disease-19 pandemics (SARS-CoV-2) [135] have emerged in humans via zoonotic transmission. The three highly pathogenic human viruses cause similar forms of atypical pneumonia with bilateral parenchymal “ground-glass” consolidative lesions that may progress to Acute Respiratory Distress Syndrome (ARDS) [136]. The frequency of such complication varies, being highest for MERS-CoV and lowest for SARS-CoV-2, suggesting important differences in disease pathogenesis. The severe cases show progressive lymphopenia with loss of both CD4+ and CD8+ T lymphocytes; massive infiltration of the alveolar walls by neutrophil and eosinophil granulocytes; and significantly elevated levels of IL-6, IL-10, IL-2, and IFN-γ, pointing to a direct correlation between the magnitude of the inflammatory response and the severity of the disease [137]. Importantly, all three viruses induce very little, if any, type I IFN [138,139,140]. Work in a mouse model of SARS suggests that the impaired IFN production is responsible for the recruitment of monocyte-macrophages and production of proinflammatory cytokines in the lung, resulting in vascular leakage and impairment of the immune response [141].

Based on available crystal structures, the PLpro encoded by SARS-CoV, MERS-CoV, and SARS-CoV-2 share a similar domain organization with “thumb”, “palm”, and “fingers” subdomains arranged together to resemble an extended right hand [107,108,109,112,142]. The Cys-His-Asp catalytic triad is located at the interface of the thumb and palm domain, with a topology similar to that found in papain. In addition to the core catalytic domain, the enzymes contain an N-terminal Ub-fold domain that is not required for catalysis but may play a role in the immunomodulatory activity of PLpro [143]. The three enzymes cleave Ub- and ISG15-reporter substrates and synthetic poly-Ub chains, and both SARS-CoV and SARS-CoV-2 exhibit weak deneddylase activity [108]. Differences in specificity and efficiency of catalysis are likely explained by subtle changes in the surface binding pockets that mediate interaction with the substrates. As a general rule, DUBs that disassemble Ub chains have surface pockets that bind the Ub moiety preceding (S1) and following (S1′) the scissile bond, whereas DUBs that recognize mono- or-polyubiquitinated substrates lack the S1′ pocket but contain one (S2) or more (S3, S4, and S5) additional pockets that can accommodate distal Ub moieties. Based on structure and mutation analysis, the preferential recognition of K48poly-Ub by SARS-CoV PLpro relies on interaction with both S1 and S2 binding pockets, where solvent-exposed hydrophobic residues within the thumb domain interact with the Ile44 patch of the distal Ub [104]. The S2 site is also involved in the recognition of the distal Ub-fold domain of ISG15 [144], although interaction with the proximal S1 pocket was shown to play a dominant role [104]. Important differences in the architecture of both the catalytic core and binding pockets of MERS-CoV PLpro correlate with overall decreased catalytic activity and capacity to recognize all types of poly-Ub chain linkages [142]. In line with the high degree of homology (83% identity), the PLpro encoded by SARS-CoV-2 resembles the SARS-CoV enzyme in the capacity to target both K48poly-Ub and ISG15 conjugates. However, changes in the palm domain were shown to improve the interaction with ISG15 [107], while mutation of a key Leu residue that engages Ub in the S2 pocket of SARS-CoV to Thr diminishes the ability to process K48poly-Ub chains [107,108,109], which results in the preference of SARS-CoV-2 for ISG15 conjugates. 

The deconjugase activity of CoV PLpro has been implicated in the downregulation of innate immune responses [145,146]. Type I IFNs and pro-inflammatory cytokines are hardly expressed or appear late in cell-culture based models of CoV infection [147], and dysregulation of the IFN response is associated with severe lung immunopathology, influx of inflammatory monocyte-macrophages, and elevated levels of cytokines and chemokines in mouse models of SARS-CoV [141] and MERS-CoV [148] infection. A similar imbalance of the host response was recently observed in SARS-CoV-2-infected patients [140]. Two lines of evidence support a key role of the viral deconjugase in these effects. First, ectopic expression of both SARS-CoV [145,149] and MERS-CoV [145,149] and more recently SARS-Cov-2 PLpro [108] was shown to inhibit innate immune signaling pathways. Second, direct evidence for the contribution of the deconjugase activity to immune evasion was obtained using recombinant MERS-CoV viruses encoding for PLpro mutants with selective loss of the deconjugase activity but preserved polyprotein cleavage. The transcription of type I IFN and IFN-stimulated genes was markedly increased in cells infected with the mutant viruses, and infected mice showed significantly increased survival rates and faster virus clearance in spite of comparable virus replication rates in the lungs [150]. 

Although the capacity of PLpro to regulate innate immune responses appears to be firmly established, the mechanism and cellular substrates involved in this effect are not well understood. The inhibition of the type I IFN by SARS-CoV PLpro was shown to correlate with impaired phosphorylation and nuclear translocation of IRF3, suggesting that signaling through TLR3 or RIG-I may be affected [145]. Surprisingly, while PLpro interacted with IRF3 both in transfected cells and in cells infected with SARS-CoV, this was dependent on the presence of the Nsp3 transmembrane domain (PLpro-TM) and neither binding nor inhibition of IRF3 phosphorylation were affected by mutation of the catalytic Cys to Ala. A possible explanation may be found in the demonstration that PLpro-TM interacts with the STING-TRAF3-TBK1 complex via binding to the STING transmembrane domain, which promotes disruption of the complex and is associated with reduced ubiquitination of RIG-I, TRAF-3, STING, TBK1, and IRF3 [105]. In addition, expression of a PLpro construct lacking the TM domain but containing the N-terminal ubiquitin-fold domain was shown to block NF-κB signaling by stabilizing phosphorylated IκBα [116]. Stabilization of IκBα may be due to deubiquitination and the consequent inhibition of proteasomal degradation, but this was not formally proven. A recent study comparing the PLpro of SARS-CoV and SARS-CoV-2 suggests that their different activity towards K48poly-Ub and ISG15 parallels the extent of inhibition of NF-kB versus IFN signaling, with the dominant de-ISGylase activity of SARS-Cov-2 PLpro being associated with a stronger decrease of ISGylated IRF3 and stronger inhibition of the IFN response [108]. However, the multiple roles of ISGylation in the inhibition or enhancement of the IFN response via targeting of RIG-I [151] or RNF3 [151] call for some caution in assessing the significance of this finding in the context of infection. It is noteworthy that silencing of ISG15 did not affect or even enhance the type I IFN response in mice infected with different RNA viruses [152,153]. It is also important to remember that ISG15 is itself an IFN target gene and that secreted ISG15 regulates the activity of various types of immune cells, including natural killer (NK) cells, dendritic cells (DC), and neutrophils [76,78]. Thus, much remains to be done to achieve a precise molecular understanding of the mechanisms by which the PLpro encoded by different coronaviruses modulate innate immunity and how this reflects in the severity of the disease. 

### 3.2. Adenovirus Deconjugases

Adenoviruses are double-stranded DNA, non-enveloped viruses with a ≈35 kD genome that codes for at least 50 different proteins expressed during the early and late phases of infection. More than forty adenovirus serotypes infect humans, causing a range of pathologies including respiratory, ocular, and gastrointestinal infections [154]. The adenovirus protease (AVP) adenain is a 25 kD protein encoded by the conserved late gene L3 [124]. The protease activity plays an essential role in the maturation of virion-associated precursor proteins, which is required for assembly of infectious virus particles and is also involved in the uncoating of incoming virions during primary infection [155]. Adenain exhibits a papain-like fold and is structurally related to the Ub-specific protease UCH-L3 and SUMO deconjugase Ulp1, with topology of the active site Cys and His residues resembling that of Ulp1 and organization of S1–S4 substrate binding pocket similar to that of ubiquitin hydrolases [86]. Recombinant adenain was shown to cleave K48tetra-Ub and ISG15 precursor peptides in vitro, and expression of the active enzymes correlated with a global decrease of poly-ubiquitinated proteins in adenovirus-infected cells. However, the cellular substrates of these activities have not been characterized. Interestingly, overexpression was associated with decreased levels of mono-ubiquitinated Histone H2A in transfected HeLa cells, suggesting a possible role of the protease in chromatin-related events or in the regulation of the DNA damage response. 

### 3.3. Herpesvirus Deconjugases

Herpesviruses are large DNA viruses with double-stranded DNA genomes ranging from 124 to 295 kb. A characteristic property of herpesviruses is their capacity to establish latent infections in certain cell types, which allows life-long persistence in the infected hosts [156]. Seven herpesviruses are important human pathogens. Herpes simplex virus-1 and 2 (HSV-1 and -2; HHV-1 and -2) and Varicella zoster virus (VZV; HHV-3) infect epithelial cells, causing cold sores or shingles, and establish latency in sensory neurons [157,158]. Human cytomegalovirus (HCMV; HHV-5) and Human herpesvirus-6 and -7 (HHV-6 and -7) infect myelomonocytic and lymphoid cells and cause mononucleosis-like syndromes in immunosuppressed patients and roseola in children [159,160]; Epstein–Barr virus (EBV; HHV-4) and Kaposi Sarcoma herpesvirus (KSHV; HHV-8) establish latency in B-lymphocytes and are associated with the pathogenesis of lymphoid, endothelial, and epithelial cell malignancies [161,162]. The establishment of latency poses a particular challenge to these viruses since it entails adaptation to different cellular environments and the consequent establishment of cell type-dependent programs of viral gene expression. In addition, transmission to new hosts is dependent on the reactivation of virus production in the face of specific and highly effective immune responses, which requires sophisticated immune evasion strategies [163]. 

The development of activity-based ubiquitin probes capable of forming covalent adducts with the catalytic Cys of DUBs [164] was instrumental for the discovery of deconjugase activity in the N-terminal fragment of the HSV-1 large tegument protein UL36 [90]. The activity is conserved in all UL36 homologs encoded by human and animal herpesviruses investigated to date [87,128,129,130,131,132], supporting the notion that the enzymes play important roles in the biology of these viruses. In spite of very low amino acid sequence similarity, sequence alignment identified relatively well-conserved Cys and His boxes, and crystal structure of the homolog encoded by the mouse cytomegalovirus (MCMV) M48 revealed a unique organization of the catalytic core, suggesting that the viral enzymes may represent a new family of deconjugases [114]. The in vitro cleavage of fluorogenic substrates and in vivo assays in cells transfected with tagged UbLs showed that the enzymes recognize comparable efficiency of K48- and K63poly-Ub [85]. In addition, a bacterial screen based on co-expression of the EBV encoded homolog, BPLF1, with UbL-GFP (green fluorescent protein) reporters revealed specificity for both Ub and NEDD8 [85].The deneddylase activity was shown to be conserved in the homologs encoded by HSV-1, HCMV, KSHV, and mouse herpesvirus MHV-68 [85], and siRNA knockdown confirmed the involvement of BPLF1 in the progressive decrease of neddylated substrates in EBV-positive cells entering the productive virus cycle [165], corroborating the notion that the deneddylase operates in infected cells under physiological levels of expression.

The strict host-specificity of herpesviruses hampers direct testing of the contribution of the deconjugases to viral pathogenesis in humans, but compelling evidence from cell culture and animal models of herpesvirus infection supports an important role of the enzymes in virus replication and pathogenesis. While deletion of the entire or large fragments of the large tegument proteins severely impaired the release of infectious virus [121], as may be expected given their essential role in the architecture of the mature virions, decreased virus yields were also observed upon infection with recombinant viruses carrying mutation of the active site Cys residue [93,95,166]. Ultrastructural analysis of cells infected with a mouse Pseudorabies virus (PrV) carrying an inactivating mutation of the pUL36 deconjugase revealed accumulation of naked nucleocapsids in the cytoplasm [166], suggesting that enzymatic activity is required for virus assembly and egress. The contribution of the deconjugase to viral pathogenesis in vivo is clearly illustrated by the strongly reduced formation of T cell lymphomas in chicken infected with mutant Marek’s disease virus (MDV) [167] and decreased neuro-invasion and longer survival of mice infected with mutant PrV [166]. Interestingly, in line with the known role of the large tegument protein in the early phases of herpesvirus infection, abrogation of the deconjugase activity was shown to cause the accumulation of incoming viral genomes in the cytoplasm of MHV-68-infected cells, which correlated with strongly enhanced activation of the type I IFN response and hampered the establishment of latent infection [168]. The capacity to interfere with the IFN response is conserved in the deconjugase encoded by human herpesviruses, as confirmed by comparing type I IFN production in cells infected with wild type and mutant viruses [89,96,102,169]. Collectively, the findings illustrate a pleiotropic role of the herpesvirus deconjugases in the regulation of multiple steps of the virus life cycle from virus entry, uncoating, and viral genome replication to the assembly and release of infectious virus particles. In addition, by halting the innate immune response, the deconjugase may promote establishment of a cellular and host environment conducive to latency and permissive for virus reactivation.

In line with the broad effect of the herpesvirus deconjugases on different cellular functions, several putative substrates have been identified, often based on candidate approaches where the capacity of the isolated enzymatic domains to deconjugate known UbL substrates was tested in co-transfection assays. While the very potent and broad deconjugase activity of the overexpressed enzymes calls for some caution in the interpretation of the data, at least some of the candidate substrates could be validated by mapping the sites of interaction and by comparing their fate in cells infected with wild type and mutant viruses. Cullins are the main cellular targets of neddylation and an obvious candidate substrate for the deneddylase activity of the EBV-encoded BPLF1 in productively infected cells [165]. BPLF1 was shown to interact with a conserved region in the C-terminal domain of the cullins scaffolds, close to the site of neddylation, and to promote cullin deneddylation and their degradation by the proteasome [85,170]. The phenotype of cells expressing catalytically active BPLF1 is similar to that induced by chemical blockade of the neddylation cascade, with accumulation of several substrates of nuclear cullin ligases and arrest in the S/G2 phase of the cell cycle [171], pointing to a role of the deconjugase in the induction of a pseudo S-phase environment that is required for efficient replication of the herpesvirus genomes [172]. In line with this possibility, viral DNA replication was strongly decreased upon siRNA-mediated knockdown of BPLF1 in cells entering the productive virus cycles, which correlated with failure to accumulate several substrates of cullin ligases, including the cellular DNA polymerase licensing factor CDT1 (chromatin licensing and DNA replication factor-1) [85]. Viral DNA replication was restored following reconstitution of CDT1 expression in BPLF1 knockdown cell, supporting the involvement of the cellular licensing factors in viral DNA replication. The DUB activity of the viral enzymes is likely to synergize with the deneddylase activity in promoting the efficiency of virus replication. Reactivation of the productive virus cycle triggers the DNA damage response [173]. In response to DNA damage, PCNA (proliferating cell nuclear antigen) is monoubiquitinated by RAD18 (RING type ubiquitin ligase-18) to activate the translesion synthesis pathway of post-replication repair. PCNA accumulates at the replication sites of many DNA viruses, although its function in viral replication is not fully understood. The EBV-encoded BPLF1 [97] and HSV-1-encoded UL36 [92] were shown to de-ubiquitinate PCNA and to prevent the formation of PCNA foci. In addition, BPLF1 was shown to interact with and to promote the accumulation of the PCNA ligase RAD18 [100] and translesion synthesis polymerase Polη [174]. Taken together, these findings point to an important role of the viral deconjugase in regulating the stability, localization and activity of a variety of cellular factors that are recruited at the site of viral replication to assist or counteract the production of infectious virus. 

Several members of the type I IFN and NF-κB signaling pathways have been proposed as putative targets of the inhibitory effect of the herpesvirus deconjugases on the innate immune responses. In different experimental setups, expression of the catalytically active enzymes was accompanied by impaired ubiquitination of RIG-I [102,169] TRAF6 [98,175,176], TRAF3 [89,176], IRAK1 [176], IRF7 [176], STING [176,177] and IκBα [88]. However, since the activity of these signaling molecules is interconnected via different ubiquitination and deubiquitination events, the identity of the viral substrates and the molecular interactions leading to failure to activate the key executor transcription factors remain in many cases unknown. In addition, since the poor amino acid sequence conservation of the N-terminal domains is likely to influence the binding properties of the viral enzymes, it is unclear whether the same or different ubiquitination events are targeted by the various member of the family. An alternative unbiased approach to the identification of putative substrates and targeted signaling pathways relies on the identification of binding partners by co-immunoprecipitation and mass spectrometry. While the huge size renders this approach technically challenging for the entire large tegument proteins, the physiological relevance of the much shorter N-terminal domain is supported by the finding that processing by caspase-1 [165], or by a yet unidentified protease [123], releases the catalytic domain of BPLF1 and UL36 to promote localization of the enzymatic activity to the nucleus. Gene Ontology analysis of protein interacting with the catalytic domain of BPLF1 showed enrichment in proteins involved in numerous cellular functions including RNA transcription and metabolism, nuclear transport, intracellular trafficking, cell cycle, and apoptosis, with major interacting hubs centering around proteasome subunits, nuclear transport proteins, and several members of the 14-3-3 family of adaptor proteins [169]. The 14-3-3 proteins are conserved molecular scaffolds expressed in all eukaryotic cells that bind as homo- or heterodimers to a multitude of functionally diverse proteins, including kinases, phosphatases, and transmembrane receptors [178,179]. Network analysis revealed that BPLF1 and 14-3-3 share a high number of interacting partners, in addition to cullins, the TRIM25 ligase that regulates the IFN response via ubiquitination of RIG-I [47]. Binding of 14-3-3 was shown to stabilize the interaction of TRIM25 with RIG-I [180], which is essential for targeting the ubiquitination RIG-I to MAVS for downstream signaling [181]. The recruitment of BPLF1 to the 14-3-3:TRIM25 complex was shown to promote activation and autoubiquitination of the ligase and was associated with failure to ubiquitinate RIG-I. Interestingly, while catalytically inactive BPLF1 induced the K48-linked autoubiquitination and degradation of TRIM25, the active viral enzyme trimmed the polyubiquitin chain to mono- or di-ubiquitin and promoted the formation of TRIM25 cytosolic aggregates decorated by the autophagy receptor p62/SQSTM1 [182]. Aggregate formation and the inhibition of IFN response were abolished by mutation of solvent exposed residues in helix-2 of BPLF1 that are also involved in the interaction with cullins [170,182], pointing to a critical role of the 14-3-3:BPLF1 interaction in the assembly of the inactivating trimolecular complex. Mapping of the interacting domains provided interesting insights on the possible mechanism of inhibition. The formation of 14-3-3 homo- or heterodimers builds a groove with two symmetrically oriented binding pockets [179]. In vitro binding assays using isolated TRIM25 domains and bacterially expressed wild type and mutant 14-3-3 and BPLF1 suggest that dimeric 14-3-3 could stabilize the trimolecular complex by simultaneously accommodating in each of the two binding pockets acidic residues located in BPLF1 helix-2 and on the tip of the TRIM25 coiled-coil domain. Docking models predict that BPLF1 would not prevent the recruitment of RIG-I but that the presence of one or two conjugated ubiquitins may hinder correct positioning of the substrate towards the E2 and may contribute to functional inactivation of the ligase [182]. The finding that the BPLF1-mediated inhibition of IFN signaling is dependent on the formation of a tri-molecular complex with 14-3-3 and TRIM25 points to TRIM25 as the primary target of the viral deconjugase. While the capacity to inhibit the ubiquitination of RIG-I and other components of the signaling cascade was previously reported [89,98,102,175], the homologs encoded by HCMV and KSHV shared with BPLF1 the capacity to bind to 14-3-3 and TRIM25 and to promote the functional inactivation of TRIM25, suggesting that this early step of the signaling cascade is a common target of the viral enzymes. Interestingly, this property was not shared by the HSV1 UL36 homolog where changes in the exposed residues of helix-2 prevent efficient binding to 14-3-3 [183]. It remains to be seen whether and how the targeting of different steps of the IFN and NF-κB signaling pathways impacts the life cycle of these viruses. 

## 4. Antiviral Compounds Targeting UbL Deconjugases

Given the growing body of evidence demonstrating the contribution of viral deconjugases to virus replication and pathogenesis, the enzymes are now recognized as attractive targets for the design of antiviral therapeutics. High-throughput screening of small-molecule libraries and rational structure-guided design have led to the identification and development of several lead compounds capable of inhibiting the activities of the PLpro of coronaviruses [116]. Among those, naphthalene inhibitors are particularly interesting due to their capacity to act as competitive, non-covalent inhibitors of SARS-CoV PLpro via interaction with a mobile loop in the fingers domain that, upon binding of the inhibitor, closes towards the catalytic cleft and shuts down the active site [103]. The compounds were shown to inhibit the deconjugase activity against model substrates and to block IFN production and virus replication in infected cells. While structural difference in the mobile loop are likely to explain the failure of the compounds to inhibit the PLpro of MERS-CoV [107,184], recent findings demonstrate potent inhibitory activity against the PLpro of SARS-CoV-2 [108]. Of note, the naphthalene compounds did not inhibit the activity of human deconjugases, caspases-3, and cathepsin-k in biochemical assays [185], which may explain their lack of toxicity in cell cultures and makes them promising candidates for the development of specific antiviral drugs. 

Both covalent and non-covalent inhibitors of the adenovirus protease working at nanomolar concentrations in biochemical assays have been described [186]. However, the compounds have either weak antiviral activity, suggesting poor cell permeability, or are highly cytotoxic for cultured cells. Thus, additional medicinal chemistry optimization will be required to harness their therapeutic potential. 

The involvement of the herpesvirus deconjugases in the regulation of both virus production and antiviral responses makes them interesting candidates for the development of much needed drugs for the treatment of many diseases caused by herpesvirus infection and reactivation. Unfortunately, research in this area is still scarce. Recently, the antiparasitic drug suramin was identified as a possible candidate in a high-throughput screen for small molecule inhibitors of BPLF1 [187]. Suramin inhibited the cleavage of K63poly-Ub at sub-micromolar concentrations and decreased in a dose-dependent manner the production of infectious virus without apparent cell toxicity. However, comparable levels of inhibition were observed against a panel of ten human DUBs, indicating that the compound has relatively poor selectivity and may be acting via a nonspecific mechanism. 

## 5. Conclusions

**Figure 4:**
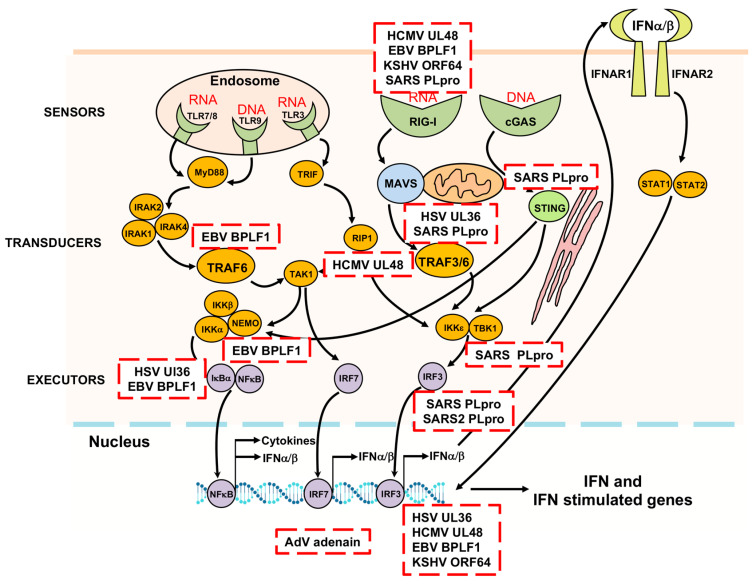
Cellular targets of viral UbL deconjugases. Viral UbL deconjugases target different steps of the innate antiviral response, ultimately leading to inactivation of the NF-κB, IRF3 and IRF7 transcription factors and inhibition of the production of inflammatory cytokines and type I IFNs. In addition, nuclear targets of the deconjugases encoded by DNA viruses regulate viral DNA replication and activation of the DNA damage response.

As regulators of both the virus life cycle and the host innate immunity, viral UbL deconjugases serve as multifunctional swiss army knives to facilitate viral infection and pathogenesis (Figure 4). Herpesviruses provide a particularly enticing example of the involvement of UbL deconjugases in the regulation of a multitude of nuclear and cytoplasmic events that are critical for the establishment of both latent and productive infection in different cell types. Since the first report on the ubiquitin deconjugase activity of adenain some twenty years ago, significant progress has been made in the identification, functional characterization, and structure determination of this fascinating class of viral enzymes. However, a precise understanding of the mechanism of action and the identification of viral and cellular substrates remain in many cases a challenge. The huge societal impact of the coronavirus pandemics is providing a strong stimulus towards the characterization of the deconjugases encoded by these viruses and the development of therapeutic inhibitors. Regrettably, work on the adenovirus and herpesvirus deconjugases is lagging behind. While the similarity to cellular enzymes is likely to be a major hinder towards the development of specific inhibitors targeting the catalytic core, the mapping of binding domains involved in substrate interaction may offer new opportunities for regulating the function of the viral deconjugases. 

## Figures and Tables

**Table 1:** UbL deconjugases encoded by human viruses.

Virus	Viral Protein	Specificity	Viral Substrates	Cellular Substrates	Ref.
Adenoviridae					
Adenovirus (AdV)	Adenain/AVP (UCH/SENP-like)	K48/K63poly-Ub, ISG15	capsid proteins	Histone H2A?	[86,87]
Herpesviridae					
Herpes simplex virus (HSV1, HHV1)	UL36	K48/K63poly-Ub, Nedd8	UL36	TRAF3, IkBa, PCNA,	[88,89,90,91,92]
Cytomegalovirus (HCMV, HHV5)	UL48	K48/K63poly-Ub, Nedd8	UL48	RIP1	[93,94,95,96]
Epstein-Barr virus (EBV, HHV4)	BPLF1	K48/K63poly-Ub, Nedd8	RR1	Cullins, PCNA, Rad6/18 TRAF6, IκBα; NEMO, TRIM25	[85,97,98,99,100]
Kaposi sarcoma herpesvirus (KSHV, HHV8)	ORF64	K48/K63poly-Ub, Nedd8	?	RIG-I	[101,102]
Coronaviridae					
Severe acute respiratory syndrome coronavirus (SARS-CoV)	PLpro (USP-like)	K48poly-Ub, ISG15	polyprotein	RIG-I, TRAF3, STING, TBK1, IRF3	[103,104,105]
Middle east respiratory syndrome coronavirus (MERS-CoV)	PLpro (USP-like)	all type of poly-Ub, ISG15	polyprotein	RIG-I	[106]
New severe acute respiratory syndrome coronavirus (SARS-CoV-2)	PLpro (USP-like)	K48poly-Ub, ISG15	polyprotein	IRF3	[107,108,109]
